# Deep Learning Techniques for Grape Plant Species Identification in Natural Images

**DOI:** 10.3390/s19224850

**Published:** 2019-11-07

**Authors:** Carlos S. Pereira, Raul Morais, Manuel J. C. S. Reis

**Affiliations:** 1 Instituto Politécnico do Porto, Escola Superior de Tecnologia e Gestão, Rua do Curral, Casa do Curral-Margaride, 4610-156 Felgueiras, Portugal; cmp@estg.ipp.pt; 2 INESC TEC/UTAD, Quinta de Prados, 5001-801 Vila Real, Portugal; rmorais@utad.pt; 3 IEETA/UTAD, Quinta de Prados, 5001-801 Vila Real, Portugal

**Keywords:** AlexNet deep model, transfer learning techniques, natural vineyard images, leaf vein extraction, independent component analysis, grape variety identification, precision viticulture

## Abstract

Frequently, the vineyards in the Douro Region present multiple grape varieties per parcel and even per row. An automatic algorithm for grape variety identification as an integrated software component was proposed that can be applied, for example, to a robotic harvesting system. However, some issues and constraints in its development were highlighted, namely, the images captured in natural environment, low volume of images, high similarity of the images among different grape varieties, leaf senescence, and significant changes on the grapevine leaf and bunch images in the harvest seasons, mainly due to adverse climatic conditions, diseases, and the presence of pesticides. In this paper, the performance of the transfer learning and fine-tuning techniques based on AlexNet architecture were evaluated when applied to the identification of grape varieties. Two natural vineyard image datasets were captured in different geographical locations and harvest seasons. To generate different datasets for training and classification, some image processing methods, including a proposed four-corners-in-one image warping algorithm, were used. The experimental results, obtained from the application of an AlexNet-based transfer learning scheme and trained on the image dataset pre-processed through the four-corners-in-one method, achieved a test accuracy score of 77.30%. Applying this classifier model, an accuracy of 89.75% on the popular Flavia leaf dataset was reached. The results obtained by the proposed approach are promising and encouraging in helping Douro wine growers in the automatic task of identifying grape varieties.

## 1. Introduction

Throughout the world, harvests within vineyards can vary from year to year with significant morphological changes on the vines, mainly due to soil conditions, diseases, pests, adverse climate, and the presence of pesticides. In addition, the vineyards of the Douro Region are also characterized by having more than one grape variety per parcel and even for row [1]. Knowing the susceptibility of a particular variety to a specific disease, its identification using this automatic system will help, for example, in a more specific and targeted treatment. In addition, many wine producers require this large number of grape varieties to produce their most expensive wines (e.g., the “Blend D. Antónia” wine, from the *Quinta do Vallado*, has more than 30 grape varieties). Consequently, implementing an automatic algorithm for grape variety identification to provide an automatic splitting of the different grape varieties will be of paramount importance in the Douro Demarcated Region.

Grape variety identification by in-field images is a challenge because the images mirror of the adverse effects caused by nature in the grapevines and by the high visual similarity of the leaf images on the different grape varieties.

To date, the literature has focused mostly on the processing of images prepared in laboratories. Usually, the objects in study were isolated at the center of the image over a white background. Every approach over images in the natural environment must be able to deal with issues about lighting conditions and occlusion of the grapes and leaves, in addition to all the previously mentioned factors.

Under these assumptions, the authors decided to use deep learning (DL), as defined by Goodfellow et al. [2]. In the area of machine learning and artificial intelligence, deep learning is recognized as a remarkably active research with successful applications in numerous fields, namely, for precision agriculture purposes. One of the strongest advantages of using DL in image classification is its powerful image feature extractor, both from raw or pre-processed data, avoiding the traditional and time-consuming process of hand feature extraction. Hand feature extraction frequently requires the intervention of an agriculturist expert and must be altered whenever the dataset or the problem changes. Deep learning automatically searches for low-level features, such as curves and edges, until higher levels of its hierarchical structure model are reached [3].

This systematic approach for the hierarchical processing of knowledge and the complex nonlinear model mark the relevance of the DL network. A convolutional neural network (ConvNet) is a DL network with many hidden layers, which attempts to imitate the way the visual cortex of the brain processes and recognizes images. ConvNet has achieved “state-of-the-art results” in image classification by providing the network with a huge number of natural images [4,5,6].

Extending this idea, richness and diversity were applied in the experiments on the pre-processed and augmented image dataset contents, which were crucial for the classification performance enhancement in a ConvNet. Because of these reasons, the AlexNet ConvNet, trained on diverse pre-processed image datasets, was used. Developed by Krizhevsky et al. [7], the AlexNet is a model pretrained on a subset of the ImageNet database which comprises more than one million images and could classify images into 1000 object categories. The AlexNet was the winner in the ImageNet Large-Scale Visual Recognition Challenge.

Reusing a pretrained model can solve the long time needed to train such very deep ConvNets. This method, called transfer learning (TL), is frequently used in the computer vision area and allows building accurate models in a timesaving way [8]. It consists of starting the learning process from patterns that have been previously learned when solving a different problem instead of starting from scratch.

Kamilaris and Prenafeta-Boldú [3] have studied the importance of verifying the conditions of the test, i.e., whether authors had tested their implementations on the same or different datasets in previous literature. From 40 research papers analyzed in the study, they concluded that only 20% of the papers used different datasets for testing and for training, having obtained accuracy results generally less than 70% in these cases. Examples of this are the works of Potena et al. [9] and Dyrmann et al. [10], reaching an accuracy of 59.4% and an IoU (Intersection over Union) segmentation metric of 0.64, respectively.

Imaged-based plant identification proposed by Grinblat et al. [11] demonstrated the benefits of using a DL approach instead of applying traditional image processing and classification techniques, which were reported in their previous research [12]. In both papers, three legume species, namely, soybeans and white and red beans, were studied based only on the analysis of their vein morphological patterns. Primarily, penalized discriminant analysis achieved the best average accuracy with 89.9%, followed by support vector machines (SVM) with linear kernel presenting 89.7% and ConvNet with five layers presenting a maximum accuracy of 96.9%. Considering that, for plant species recognition, leaf veins contain important information, Fu and Chi [13] proposed an approach to extract leaf veins that combined a thresholding method and an artificial neural network (ANN) classifier. They also developed a preliminary segmentation based on the intensity histogram of leaf images, followed by a fine segmentation using a trained ANN classifier. Experimental results showed that this approach was able to extract more accurate venation of the leaf for vein pattern classification.

Zernike moments and color information for the description of the grape shapes were also exploited concerning grape detection in outdoor images. For the learning and recognition steps, an SVM was used. In 99% of cases, the recognition of grapes was successful, with very few samples misidentified [14]. From a dataset of 190 images containing bunches of white grapes and 35 images containing red grapes, the visual inspection system using color mapping and morphological dilation techniques, proposed by Reis et al. [1], was able to automatically distinguish between red and white grapes and achieved 97% and 91% of correct classification, respectively.

Kho et al. [15] proposed an automated system to identify three species of Ficus (reaching about 1000 species worldwide, one of the largest genera in plant kingdom) with similar leaf morphology. Fifty-four leaf images from three different Ficus species were used. Image pre-processing, feature extraction, and recognition models, using ANN and SVM, were carried out to develop the proposed system. Evaluation results reached an accuracy of 83.3%, demonstrating the ability of the proposed system to recognize leaf images.

From a different perspective, Reiser et al. [16] proposed the use of a 3D imaging system using a sonar sensor and an automated three-axes frame for selective spraying in controlled conditions. Within these conditions, a robot was able to automatically detect the positions of the plants with an accuracy of 2.7 cm and was also able to spray on these selected points. Additionally, this selective spraying reduced the used liquid by 72% when compared to a conventional spraying method in the same conditions. The stem position and plant height of reconstructed maize plants were determined by Vázquez-Arellano et al. [17] using a low-cost time-of-flight camera. They merged four-point clouds generated from different 3-D perspective views using the iterative closest point algorithm. The plant stem positions were estimated with an average mean error of 24 mm and standard deviation of 14 mm. The individual plant height estimation average mean error was 30 mm, and standard deviation was 35 mm. The overall plant height profile average mean error was 8.7 mm.

Nuske et al. [18] proposed a vision system that automatically predicts yield in vineyards accurately and with high resolution. As stated by the authors, “The system incorporates cameras and illumination mounted on a vehicle driving through the vineyard”. They exploited three prominent visual cues of texture, color, and shape into a strong classifier that detects berries, and maximized the spatial and the overall accuracy of the yield estimates by optimizing the relationship between image measurements and yield: “Analysis of the results demonstrates yield estimates that capture up to 75% of spatial yield variance and with an average error between 3% and 11% of the total yield”.

Studies of ConvNet applications for image-based plant disease classification were recently published [3,19,20,21,22,23]. Ferentinos [20] used simple leaf images of healthy and diseased plants from an open database of 87,848 images containing 25 different plants in a set of 58 classes of plant and disease combinations. An accuracy score of 99.53% was reached, making the author’s approach promising to operate in real cultivation environment. Fuentes et al. [21] presented a DL-based approach to detect diseases and pests in tomato plants from natural images. Experimental results demonstrated that the authors could recognize nine different types of diseases and pests, reaching accuracies of 55.64% and 83.06% without and with data augmentation, respectively. Mohanty et al. [23] developed a smartphone-aided disease diagnosis system. A public image database of 54,306 images of diseased and healthy plant leaves was used for training DL models. Using a held-out test set to calculate the systems performance, the best trained model reached an accuracy of 99.35%.

Research works of DL for grape recognition and segmentation were recently proposed. Milella et al. [24] developed methods for automated grapevine phenotyping, namely, the canopy volume estimation and detection and counting of grape bunches. The authors used four ConvNet architectures (the AlexNet, VGG16, VGG19, and GoogleNet) to compare segment visual images, acquired by a task-specific sensor, into multiple classes and recognized grape bunches. According to the authors, the proposed methods, tested in-field for fruit detection, achieved a maximum accuracy of 91.52% with the VGG19 model. A method for automatic grape bunch detection in color images was developed by Marani et al. [25]. They used a pretrained ConvNet to perform image segmentation. Classification in a few classes of interest was made and compared with sub-patches of known size. Probability maps for each class were applied for pixel-by-pixel segmentation of the grape clusters. An accuracy of 87.5% was reached to segment grape bunches in-field images.

This investigation evaluated the accuracy levels for the identification of the grape varieties of the Douro Demarcated Region in natural vineyard images using several Transfer Learning (TL) schemes on the AlexNet ConvNet, which was trained using diverse pre-processed image datasets and data augmentation methods. A proposed image warping method, named four-corners-in-one, accompanied by the proposed leaf segmentation algorithm (LSA), demonstrated success achieving the best classification accuracy in the set of performed experiments.

## 2. Materials and Methods

**Figure 1:**
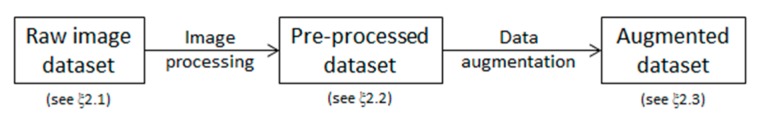
Block diagram for the generation of an augmented pre-processed dataset.

At all stages of image classification or object recognition research, appropriate datasets are required, beginning with the training phase on the CNN-based models to evaluate the performance of any classification or recognition algorithms [19]. Figure 1 presents a block diagram for the generation of the augmented pre-processed datasets.

### 2.1. Original Raw Datasets

In this work, we collected two natural vineyard image datasets at the Douro Demarcated Region over two years: (i) In the 2016 harvest season, an image dataset named Douro Red Grape Variety was collected (DRGV), and (ii) in the 2018 harvest season, an image dataset named DRGV_2018 was collected. 

**Figure 2:**
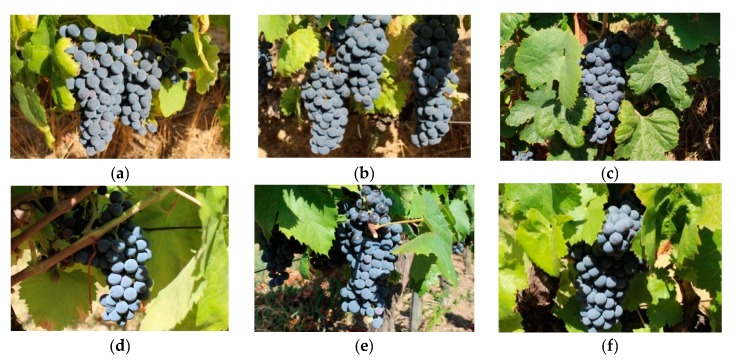
A sample of each one of the six red grape varieties of the Douro Red Grape Variety (DRGV) database: (**a**) *Touriga Nacional*, (**b**) *Tinta Roriz*, (**c**) *Touriga Franca*, (**d**) *Tinto Cão*, (**e**) *Tinta Barroca,* and (**f**) *Tinta Amarela*.

**Figure 3:**
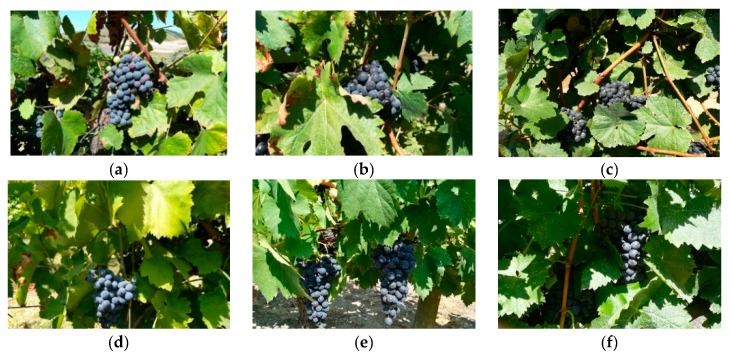
A sample of each one of the six red grape varieties of the DRGV_2018 database: (**a**) *Touriga Nacional*, (**b**) *Tinta Roriz*, (**c**) *Touriga Franca*, (**d**) *Tinto Cão*, (**e**) *Tinta Barroca*, and (**f**) *Tinta Amarela*.

The DRGV dataset was previously described by Pereira et al. [26] and comprised 140 vineyard images. It possessed six different red grape varieties, namely, *Tinta Amarela, Tinta Barroca, Tinto Cão, Touriga Franca, Touriga Nacional,* and *Tinta Roriz*, with nearly 23 images per variety. The DRGV_2018 dataset comprised 84 vineyard images, also distributed by the same six grape varieties with an average of 14 images per variety. The characterization of the two raw datasets is summarized in Table 1. Figure 2 and Figure 3 present examples of one image per grape variety for the DRGV and DRGV_2018 datasets, respectively.

The DRGV dataset was captured in the 2016 harvest season, during a full-sun day in August, using a CANON EOS 600D camera with a resolution of 5184 × 3456 pixels and image aspect ratio of 3:2 (Canon Inc., Ōta, Tokyo, Japan). The DRGV_2018 dataset was captured in the 2018 harvest season, during a full-sun day in August, using a HUAWEI P8 lite 2017 smartphone equipped with a 12 MP BSI camera, a resolution of 3968 × 2976 pixels, and image aspect ratio of 4:3 (Huawei Technologies Co. Ltd., Shenzhen, Guangdong, China).

Given that our raw datasets were very small, the DRGV_2018 dataset was replaced by merging it with the DRGV dataset, named DRGV + 2018, summing 224 images distributed by the six different grape varieties with an average of 37 images per variety. In summary, this work was developed taking into account two effective raw datasets: The DRGV and the DRGV + 2018 datasets. 

### 2.2. Datasets and Pre-Processing

In a recent DL survey, Kamilaris and Prenafeta-Boldú [3] reported some pre-processing methods used, including image resizing, segmentation, background removal, foreground pixel extraction, and features extracted from the images, such as shape, statistics, histograms, principal components analysis filters, wavelet transformation, and gray-level co-occurrence matrix features, among others.

In the present study, a first collection of seven pre-processed datasets (#1 to #7), extracted from the DRGV dataset, and a second collection of three pre-processed datasets (#8 to #10), extracted from the DRGV + 2018 dataset, were generated.

**Figure 4:**
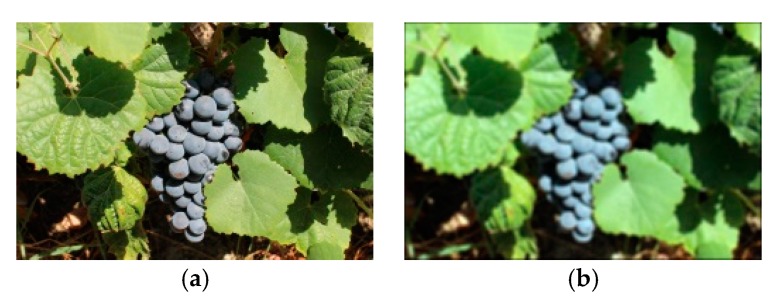
Independent component analysis: (**a**) DRGV image; (**b**) Convolution output of (**a**) with the first independent components filter (ICF).

The first collection encompassed diverse image processing (IP) methods. The first IP method, independent component analysis (ICA), is a signal processing technique which obtained interesting results in natural images in previous research [27,28,29], prompting its use as a pre-processing technique for grape variety identification in natural environment in this study. For grape variety identification, the popular fixed-point FastICA algorithm, developed by Hyvärinen and Oja [30] and based on the maximization of the kurtosis, was used, which generated all independent components filters (ICFs). Figure 4 depicts the convolution output of an image with the first ICF.

The next two IPs, the Canny edge detector (CED) proposed by Canny [31] and gray-scale morphology processing (GMP) developed by Zheng and Wang [32], are related with the leaf vein extraction. Leaf venation aims at identifying the vein pattern of a plant species. The primary vein is similar to the main trunk of a tree, while the secondary vein is similar to the major limbs of a tree [13].

In a leaf image, the leaf vein can be seen as noise on the leaf surface and displays distinguishable patterns between species. Methods of noise detection, like mathematical morphology, may be useful for leaf vein extraction. Thus, Zheng and Wang [32] proposed a new method for leaf vein extraction based on GMP, which basically comprised two basic mathematical morphology operations: Erosion and dilation. Other morphology operations, such as the opening and closing operations, the bot-hat transformation, and the top-hat transformation, were produced based on different combinations on these two basic operations. 

In our case, the complete GMP was composed of six morphology operations, designed according by Equation (1).

**Figure 5:**
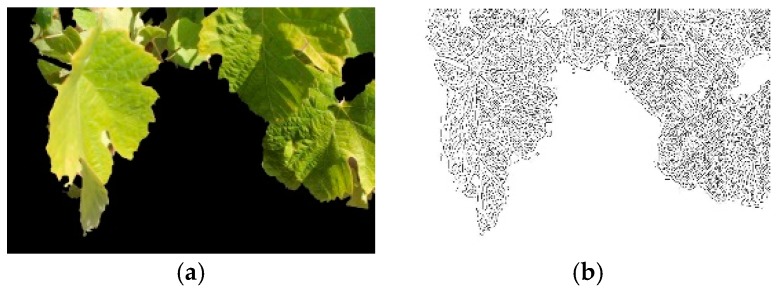
Canny edge detection operator: (**a**) Segmented leaf image; (**b**) Canny output result.

**Figure 6:**
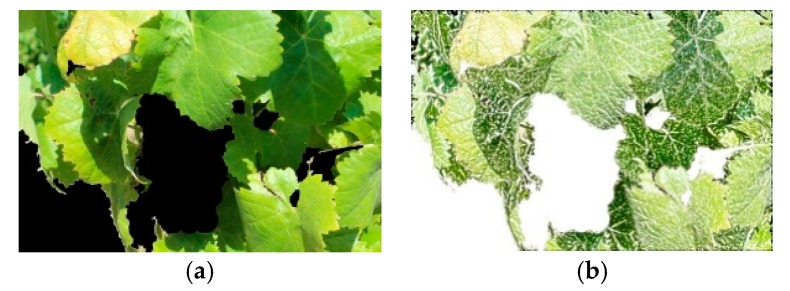
Gray morphology procedure: (**a**) A segmented leaf image; (**b**) Gray morphology output.

(1) g=(f·b−f)−(f−f∘b)

where *f* is a gray image, *b* a structuring element, f∘b is an opening operation, f·b is a closing operation, f−f∘b is a top-hat transformation, f·b−f is a bot-hat transformation, and *g* is the gray image output. The selected structuring element *b* was a square with a width of seven pixels, with an experimentally obtained value. A sample of the CED and GMP is shown in Figure 5 and Figure 6, respectively.

**Figure 7:**
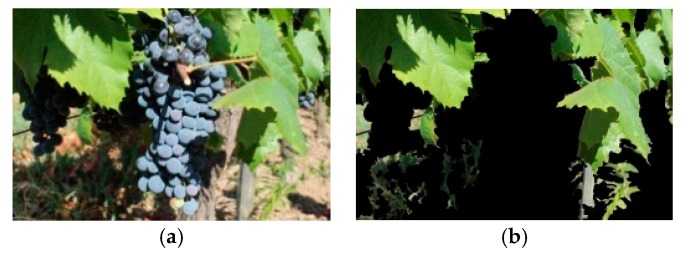
The proposed method for grapevine leaf segmentation: (**a**) An original image from the DRGV database; (**b**) segmented leaf output.

A proposed four-corners-in-one image warping method, described next, was used. From an original raw image (Figure 7a), the LSA, proposed by Pereira et al. [26], was applied. The segmented leaf image is shown in Figure 7b.

**Figure 8:**
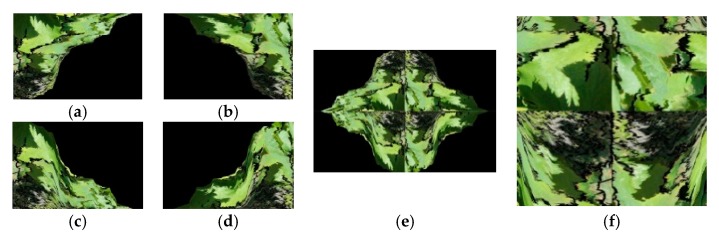
The steps of the proposed four-corners-in-one method: (**a**) Northwest corner; (**b**) northeast corner; (**c**) southwest corner; (**d**) southeast corner; (**e**) images (**a**–**d**) joined after a rotation of 180° of each image; (**f**) larger square image patch extracted from (**e**), with non-black pixels inside it (enlarged view for better visualization).

All non-black (color) pixels of the image presented in Figure 7b were concentrated at the northwest corner of the image. For image processing, a sequence of a left shift, followed by a sequence of up shift operations of the colored pixels of the image, were performed (Figure 8a).

The algorithm used for processing the northwest corner was replicated for the other three corners of the image by the sequence: Northeast, southeast, and southwest, replacing the shift left operation by right shift and up shift operation by down shift at the east and south directions of the image, respectively. Figure 8b–d shows the resulting images. Next, the four images were concentrated in a new output image by rotating each image by an angle of 180° joining each image following the position given by the cardinal points of the images, as Figure 8e illustrates.

An auxiliary one-pixel-size white row and column, shown in the Figure 8e, which delimited the four corners of the image, were used in the algorithm to help the extraction of the larger square image patch with non-black pixels inside. Figure 8f presents the resulting image patch centered at the cross point of the white row and column. Afterward, the white row and column were removed.

The second collection of pre-processed datasets only comprised images resulting from the application of the four-corners-in-one algorithm.

### 2.3. Pre-Processed Dataset Augmentation

The aim of applying augmentation is to increase the number of objects in the dataset and introduce distortion effects to the images, which help in reducing overfitting during the training phase [19].

In this work, we generated “the fake samples” (so-called by Zhang et al. [33]) based on the training/validation set in three different means. The first data augmentation method was the one-pixel image translation with a given factor [4,7]. The factor was an integer number of generated sub-sample images. For example, the factor of 900 (F900) means that the image was translated in a range of 30 × 30 pixels both horizontally and vertically. Thus, we created new samples with size of 900 times for the training/validation set. The second augmentation method was a horizontal image reflection (mirror). For the second augmentation, the image was rotated by an angle of 180°. Finally, the third augmentation method was the image rotation (rotate). The image was transformed by the rotation angle in a range from −5° to +5° with a gap of 1° (excluding the 0°). Thus, we created new samples with size of 10 times for the original data.

**Figure 9:**
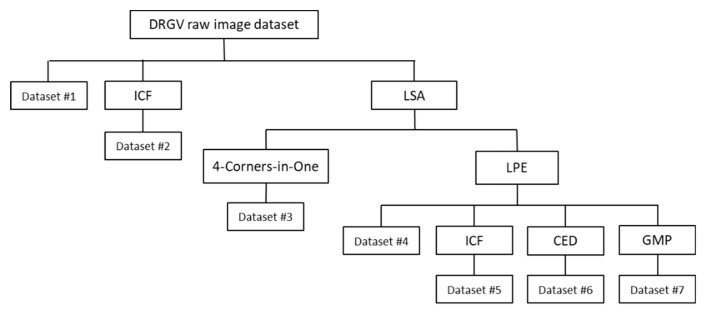
Flowchart of the pre-processed DRGV dataset generation process.

Primarily, as mentioned in Section 2.2, a collection of seven pre-processed datasets was extracted from the original DRGV dataset. The generation process of pre-processed datasets #1 to #7 is shown in the flowchart of Figure 9.

Dataset #1 was generated directly from the original raw image dataset, i.e., without applying any pre-processing method. To increase the number of training images, each image was resized to a fixed size of 256 × 256 and distinct 900 sub-images (F900) of 227 × 227 pixels were randomly cropped.

Dataset #2 was generated from the ICF convolutions. First, the matrix X, as the input to FastICA algorithm, was constructed by converting each color image to grayscale and resized to 636 × 960 pixels to accommodate 53 × 80 = 4240 non-overlapping image patches of fixed size of 12 × 12 pixels. Next, the output matrix X with 144 rows and 4240 columns was fed to the FastICA algorithm to calculate the 144 ICFs through its output variable W. Then, 25 ICFs were randomly selected (i.e., the rows of W), corresponding to the first three, last three, and the remaining 19 ICFs uniformly spaced using a gap of three filters. Each selected ICF was convolved with each one of the three channels of the original color image. Last, eight image blocks of 228 × 228 were selected, with each image block generating four images (F4) of 227 × 227 pixels.

Dataset #3 was generated from the proposed four-corners-in-one image warping method, as described in Section 2.2. Each output image was resized to 256 × 256 sub-images of 227 × 227 pixels. This increased the number of training images by F900.

Datasets #4 to #7 were generated from the segmented leaf images using the LSA. From these segmented images, the leaf patch extraction (LPE) of a set of non-overlapping 64 × 64 leaf image patches with no black pixels was performed and 3644 image patches over the DRGV dataset were cropped, forming the dataset #4. For augmentation purposes, each 64 × 64 patch was resized to 231 × 231 to extract 25 sub-images (F25) with a fixed size of 227 × 227 pixels.

Dataset #5 was generated from the previous 3644 leaf image patches using ICF convolutions. First, the matrix X, as the input to FastICA algorithm, was constructed by converting each color image to grayscale using the HSV color conversion model. Each image was then divided into 64 distinct image blocks, each with a fixed size of 8 × 8 pixels. Next, the resulting matrix X with 64 rows and 3644 columns was given as input to the FastICA algorithm to calculate all the 64 ICFs through the output variable W. Then, 25 ICFs were randomly selected (i.e., the rows of W) corresponding to the first three, last three, and the remaining 19 ICFs uniformly spaced using a gap of three filters. Each selected ICF was convolved with each one of the three channels of the original color image. Last, each 64 × 64 image patch was resized to 231 × 231 pixels to extract 25 sub-images (F25) with a fixed size of 227 × 227 pixels.

Dataset #6 was also generated from the 3644 leaf image patches. Primarily, each patch was transformed to grayscale and a Canny edge detector with a sigma factor of 1.75 was applied. All eight-connected regions containing only one pixel are set to zero. The output binary images were converted to color images by simply copying the binary channel three times. Each 64 × 64 edge image patch was resized to 231 × 231 pixels to extract 25 sub-images (F25) of 227 × 227 pixels. 

Dataset #7 was generated from the 3644 leaf image patches. It was inspired by the work developed by Zheng and Wang [32] for leaf vein extraction. For data augmentation purposes, each image patch was resized to 231 × 231 pixels to extract 25 sub-images (F25) with a fixed size of 227 × 227 pixels. 

Table 2 characterizes each one of pre-processed datasets #1 to #7, focused on the pre-processing and data augmentation methods.

**Figure 10:**
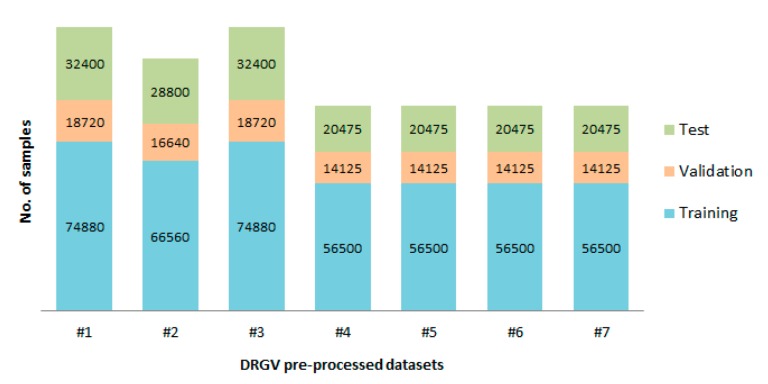
Data splitting from DRGV pre-processed datasets #1 to #7.

First, from each DRGV pre-processed dataset, six images per grape variety and their augmented forms were randomly removed for test purposes. In pre-processed datasets #1 to #7, a shuffled data splitting of 80% and 20%, for training and validation datasets, respectively, was applied. Figure 10 shows the distribution of the number of training, validation, and test samples on the DRGV pre-processed datasets.

Pre-processed datasets #8 to #10, extracted from the DRGV+2018 dataset, were generated using the proposed four-corners-in-one image warping method. Initially, 10 images per grape variety were randomly removed from 224 raw images of the DRGV + 2018 dataset for testing purposes. From the remaining 164 images, six images per grape variety were randomly removed to form a validation set (hold-out data). So, the data splitting of the DRGV + 2018 dataset was constituted by the training set (128 images, 57%), validation set (36 images, 16%), and test set (60 images, 27%).

To create the dataset #8, the 224 original raw images were resized to a fixed size of 256 × 256 pixels, in which a one-pixel image translation method with F900 (30 × 30) was applied. To produce dataset #9, each raw image was transformed by a one-pixel image translation method with F900, followed by a horizontal reflection image transformation (mirror). To construct dataset #10, each raw image was resized to a fixed size of 236 × 236 to apply the one-pixel image translation method with F100 (10 × 10), a horizontal reflection image transformation, and a rotation transformation (rotate), with angles ranging between −5° and +5° with a gap of 1°. Table 3 shows the amount of augmented training/validation and test sets for the second collection of the pre-processed datasets extracted from the DRGV + 2018 dataset.

### 2.4. Convolutional Neural Network

The ConvNet is composed of a deep structure, consisting of alternating convolution and pooling layers, and last, by fully connected layers. It has shown better results than state-of-the-art classifiers, such as SVM and linear regression, among others [34,35].

A convolution layer is defined by the number of filters (for example, the number of output channels), the properties of these filters (for example, number of input channels, width, and height of the image) and the properties of the convolution (for example, padding and stride).

The discrete convolution operation *C*, between an image *f* and a filter *g*, at the point of coordinates (*x*,*y*), is defined by Equation (2) as (see, for example, [2]):

(2) $$C\left(x,y\right)=f\left(x,y\right)⋇g\left(x,y\right)=\underset{n}{\sum }\underset{m}{\sum }f\left(n,m\right)g\left(x-n,y-m\right)$$

where ⋇ denotes the dot product between the image *f* and the filter *g* (*f* and *g* having the same dimensions). In the case of neural networks, the output matrix is typically called “a feature map”, and after processing, the activation function is “an activation map”. The output size decreases slightly with every convolution if the image *f* is not padded [2].

#### 2.4.1. Pooling and Stride

Decreasing the activation map size present at the end of the deep network makes it more suitable for classification purposes. 

A pooling layer provided invariance to marginally different input images and reduced the dimension of the feature maps (e.g., width and height). To each feature map *c*:*a* = *f*(*c*), a nonlinear function *f*( ) was then applied element-wise. The resulting activations *a* were then passed to the pooling layer. This aggregated the information within a set of small local regions, *R*, producing a pooled feature map *s* (of smaller size) at output. If *pool*( ) denotes the aggregation function for each feature map *c*, the pooled feature map is given by Equation (3) [36]:

(3) $$s_{j}=pool\left(f\left(c_{i}\right)\right)\;\forall_{i}\in R_{j}$$

where *R_j* is the pooling region *j* in the feature map *c*, and *i* is the index of each element within it.

The other way to reduce the size of the activation map is to adjust the stride parameter of the convolution operation. The convolution output can be calculated for a fixed square grid centered on every pixel of the input image (stride 1) or jumping by every *n*th pixel (stride *n*).

#### 2.4.2. Rectified Linear Unit

The layered structure of a ConvNet typically includes one or more nonlinear activation functions, the so-called rectified linear activation technique. The output of a nonlinear activation function is related to the capacity of the neural network to approximate nonconvex functions. Every type of activation function performs a certain fixed point-wise operation on a vector. The most-used *ReLU* function in ConvNet models is defined by Equation (4), where *x* is an input real number:

(4) $$ReLU\left(x\right)=\{\begin{array}{cc} x & x\geq 0\\ 0 & x<0 \end{array}$$

Due to the linear non-saturating form, the *ReLU* function greatly accelerates the convergence of stochastic gradient descent compared to the sigmoid/TanH functions. Another advantage is related to the lower computational processing effort compared to the calculation of an exponential function. However, the does not appear *ReLU* suited for all datasets and architectures because it removes all the negative information.

### 2.5. Network Structure

In this work, the structure of the AlexNet model, as proposed by Krizhevsky et al. [7], was used. The architecture of this ConvNet was pretrained on a subset of the ImageNet database, which comprises more than one million images and could classify images into 1000 object categories and 25 layers. The first 22 layers consisted of five convolutional and three max-pooling layers with different square kernel sizes and strides. Moreover, two layers of dropout with probability of 50% were linked after each fully connected layer. Next, a softmax layer was present to obtain a probability distribution, and finally, a classification layer chose the highest probability as its predicted class. Table 4 shows the details of each AlexNet layer available in the DL Matlab^TM^ toolbox (The MathWorks Inc., Natick, MA, USA).

### 2.6. Training Setting

The pretrained ConvNets are usually composed by two basic parts: The convolutional base, which performs feature extraction, and the classification base, which classifies the input image based on the features extracted by the convolutional base. Focused on the classification part, different approaches are followed to build the classifier. First, a stack of fully connected layers, followed by a softmax activated layer, are used [7,37,38]. Second, the linear SVM classifier [39] may be successfully trained on the features extracted by the convolutional base [40].

Traditional DL architectures took advantage of the transfer learning, which increases learning with the already existing knowledge of some related task associated with the problem under study by fine-tuning pretrained models [3] and builds accurate models in a timesaving way [8]. Specifically, this investigation focused on transferring the pretrained AlexNet architecture to the specific task of grape variety identification in natural vineyard images. Applying the knowledge of the TL to our application domain, fine-tuning and fixed feature extractor schemes were used, as described by Bunrit et al. [41].

To fine-tune the AlexNet to our specific task, some parts of the pretrained network were retrained, with the transferred weights and bias from the pretrained network using all the pre-processed datasets. In opposition to fine-tuning, the pretrained weights and bias of the AlexNet extracted through the fixed feature extractor scheme were directly transferred to a multiclass SVM classifier for training/validation with our pre-processed datasets.

Operationally, the classification part of the AlexNet (the last three layers) was removed and replaced by a new one that fit the six grape varieties, according to the following TL strategies: (i) “Feature (FE) and classification part”, where no convolutional layers were frozen (a frozen layer does not change the weights during training), therefore, the network was retrained using the learned features extracted from the ImageNet database and the new classification part that fits the six grape varieties identification; (ii) “Learning on last 3 conv. layers”, which involved freezing the first two convolutional layers, only setting their learning rates to zero. This implies that the network was only retrained on the last three convolutional layers and the new classification part; (iii) “FE and SVM”, where the entire convolutional base was frozen and its original activation map was then fed with a multiclass SVM classifier.

The AlexNet models were retrained for six and 30 epochs. An epoch consists of a complete training cycle over all training image sets. As usually happens in DL, the training phase takes a long time. Therefore, the creation of checkpoints to save a snapshot of the trained model parameters (weights and bias of the trained layers) when finished every epoch during training is a good practice. The checkpoints can be used as classifiers, starting points for ongoing training, or to tune the hyperparameters at any given epoch. In this work, the checkpoint/restart technique available in Matlab^TM^ was mainly applied to make and evaluate predictions on test datasets after completing each epoch.

On the training phase, a global base learning rate of 1 × 10^−5^ was used. To prevent continuous increase of validation loss during the training phase, the early stopping method was used to finish the training phase when the validation loss increased along a given number of validation points (patience parameter set to ten) that occurred at fixed-frequency intervals (set to one epoch). Furthermore, the SGDM optimization algorithm was selected, with momentum of 0.9, weight decay (L2 regularization) value of 1 × 10^−1^, and batch size of 32.

The multiclass SVM classifier was trained on the features extracted by the fully connected (fc6) layer. Ten-fold cross-validation to estimate the error of the classifier was used. Since 10-fold cross-validation was applied on pre-processed datasets #1 to #10, no data splitting into training/validation sets was needed. To optimize the hyperparameter choice of the penalty parameter C of the error term, a grid search method was used. This method is applied when multiple input parameters exist and is intended to find the area that contains the best combination of parameters. A grid search algorithm is commonly guided by some performance metric, typically measured by cross-validation on the training set. 

These parameters were determined experimentally according to the best classification accuracies on a validation set using the grid search method for diverse learning rate and weight decay hyperparameter values.

Last, to visualize the features learned by the proposed network, the *deepDreamImage* Matlab^TM^ function was used. This algorithm is based on a feature visualization technique in DL, called DeepDream, developed by Google^®^ (Google Inc., Mountain View, CA, USA) in 2015, and implemented in a computer program using a ConvNet, which synthesizes images that strongly activate the network layers.

## 3. Experimental Results

A set of experiments led to an effective study of the behavior of the TL on the AlexNet architecture trained over diverse pre-processed datasets, with the goal to achieve the best classifier for grape variety identification. So, an experimental strategy to evaluate the performance of a grape variety identification system was proposed.

The first set of experiments, using pre-processed datasets #1 to #7 (Section 2.3) trained on three different AlexNet-based TL schemes (Section 2.6), was performed. From its performance results, the effective IP method related to the pre-processed dataset that present better test accuracy was selected. The second set of experiments, using pre-processed datasets #8 to #10 (Section 2.3), was trained on the same three AlexNet-based TL schemes (Section 2.6). Section 3.1 and Section 3.2 describe the performance results obtained on the DRGV and the DRGV+2018 test datasets, respectively.

All experiments described hereafter were developed on the Matlab^TM^ 9.6 (R2019a) programming platform, using the deep learning Matlab^TM^ toolbox and running on a computer installed with Windows 10 Home, Intel Core^TM^ processor i7-7500U at 2.70 GHz, 8 GB DDR4 and dedicated graphics NVIDIA GeForce 920MX with 2 GB memory (AsusTek Computer Inc., Beitou District, Taipei, Taiwan).

### 3.1. Performance on the DRGV Test Set

In this first set of experiments, DRGV pre-processed datasets #1 to #7 were trained using the data splitting scheme, shown in Figure 10. From each DRGV pre-processed dataset, six images per grape variety and their augmented forms were randomly removed for test purposes. The AlexNet models were retrained for six epochs.

Table 5 shows the training, validation and test accuracy, and loss values obtained by different TL schemes trained on the DRGV pre-processed datasets. The IP method which reached better performance results on the training, validation, and test datasets was the proposed four-corners-in-one image warping method, as depicted in Table 5. 

### 3.2. Performance on the DRGV + 2018 Test Set

In this second set of experiments, pre-processed datasets #8 to #10 were trained using the data splitting scheme, shown in Table 3. To evaluate the performance of pre-processed datasets #8 to #10 on the training progress, the training and validation datasets were shuffled. From each pre-processed dataset, 10 images per grape variety and their augmented forms were used for test purposes. The AlexNet models were retrained for 30 epochs.

The performance results obtained from pre-processed datasets #8 to #10 are depicted in Table 6, where every column named “epoch” indicates the number of the epochs (or n/a, meaning “not applicable”) where the test accuracy was higher. In Table 6, the maximum testing accuracy of 77.30% using the “Learning on last 3 conv. layers” TL scheme of the AlexNet model to train the network from pre-processed dataset #9 is highlighted. Using this deep model trained on the original raw image dataset, i.e., without applying any pre-processing method, a classification accuracy of 76.01% was achieved on the test set. This result highlights the relevance of the application of the four-corners-in-one image warping method on the original dataset for grape variety identification.

The confusion matrix over the test set was constructed (Table 7). The grape variety *Touriga Franca* was identified with the highest accuracy of 89.1%. The varieties with worst performance results were the *Touriga Nacional*, *Tinta Roriz,* and *Tinto Cão*, presenting accuracies of 65.65%, 67.1%, and 72.95%, respectively. From the confusion matrix in Table 7, the worst case of misclassification occurred with the *Tinta Roriz* variety images, of which 3417 (18.97%) images were misclassified as *Touriga Franca*.

The proposed grape variety identification system comprised a deep classifier using the pretrained AlexNet architecture and trained on a raw image dataset pre-processed with the proposed four-corners-in-one image warping method. 

### 3.3. Network Feature Visualization

The two-dimensional filters applied on the successive convolutional and fully connected layers of a ConvNet can be visualized through the images that highlight the types of features that the network will detect. The visualization of the convolutional features in the several layers of a ConvNet can help to understand what kind of pattern a certain filter might detect and is useful to evaluate the progress of the training phase. Usually, a well-trained network exhibits well-formed, smooth feature images with an absence of noise.

**Figure 11:**
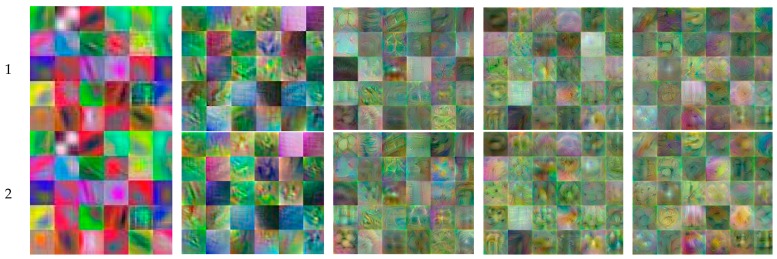

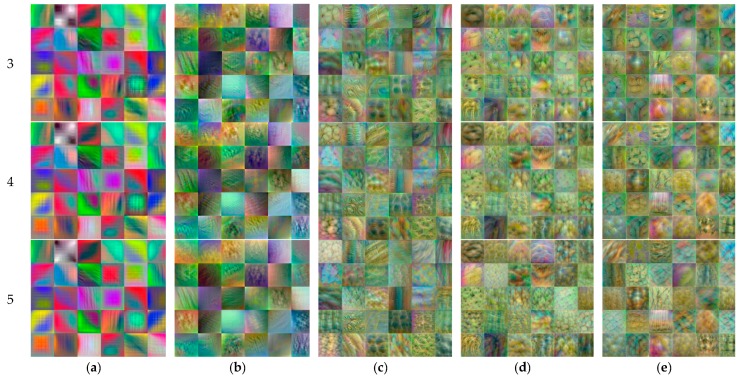
The first 30 features learned by the five convolutional layers of the proposed classifier (Leftmost column: *PyramidLevels* values): (**a**) Conv1; (**b**) Conv2; (**c**) Conv3; (**d**) Conv4; (**e**) Conv5.

Figure 11 shows the first 30 features learned by the five convolutional layers of the proposed classifier, trained over all the images of pre-processed dataset #9, which was visualized in different image detail levels. The *deepDreamImage* function with the *PyramidLevels* parameter enabled us to produce more detailed images. Setting this parameter to 1, the images were not scaled.

The feature images produced by the first two convolutional layers (Figure 11a,b) contained edges and colors, which indicates that the earlier layers learned about basic features in images. Moving deeper into the last three convolutional layers, it became difficult to interpret the patterns because the deeper layers learned much more abstract information regarding more complex features, which led to generalizations about the classes and not about the own characteristics of the image.

**Figure 12:**
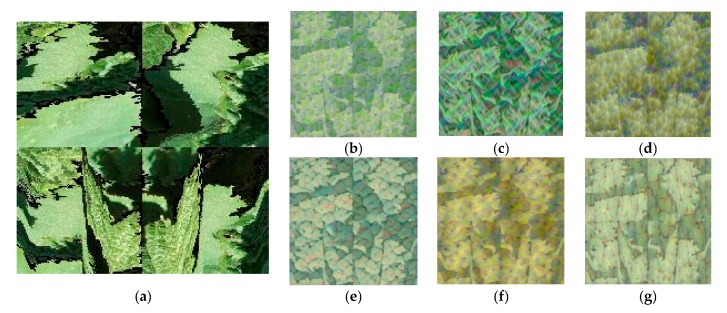
The six features learned by the last fully connected layer (fc8) of the proposed network when identifying an image of *Touriga Franca* displayed in: (**a**) Test image, as variety of: (**b**) *Tinta Amarela*, (**c**) *Tinta Barroca*, (**d**) *Tinto Cão*, (**e**) *Touriga Franca*, (**f**) *Touriga Nacional*, and (**g**) *Tinta Roriz*.

To produce feature images that resemble a given class, the final fully connected layer was selected. The fully connected layer, toward the end of the ConvNt, learned the high-level combinations of the features from the earlier layers. Figure 12 depicts the six features (six grape variety classes) by the final fully connected layer of the proposed classifier network when identifying the class of the test image, belonging to the grape variety *Touriga Franca*, shown in Figure 12a.

## 4. Discussion

Leaves play an important role in plant identification and plant disease recognition, mainly because they are easily found and captured in fields during the vegetation period. In the last few years, several researchers have studied automatic plant identification [11,15,42] and plant disease detection [16,17,18,19,20,21,22,23,43,44,45] from leaf images using DL models, achieving very high classification accuracies. However, the following three situations may lead to these results.

First, it is important to verify if the researching authors tested their implementations using the same dataset (e.g., by dividing the dataset into training and testing/validation sets) or using different datasets to test their solution. Most of the released papers did not use different datasets for testing and for training/validation [3].

Second, many other authors have obtained excellent performance results on the previously mentioned challenging agricultural problems using DL networks in the range of 99–100% in publicly available datasets of plants or leaves. However, in images captured in natural environments, these accuracy values were much lower. Examples of this are the works of Potena et al. [9] and Dyrmann et al. [10], reaching an accuracy of 59.4% and an IoU (Intersection over Union) segmentation metric of 0.64, respectively.

The third situation, reported by Šulc and Matas [46], presented excellent performance results when ConvNets were applied on sufficiently large datasets. In different plant recognition experiments, when extensive training data is available, better accuracy can be achieved using a ConvNet, performing leaf classification almost perfect with 99.9–100% accuracy on the MEW (Middle European Woods) dataset with 153 plant species. Although the experimental results obtained by these authors suggest that the “recognition of segmented leaves is practically a solved problem, when high volumes of training data are available”, they also concluded that, in the presence of a small number of samples, the identification problem remains a valid problem for uncommon plant species and rare phenotypes, among others. To try to solve this problem, a higher volume of natural images, captured along various harvest seasons in different geographical locations at the vineyards of the Douro Demarcated Region, should be acquired.

The long time needed to train DL networks is one of the main drawbacks of using this methodology of image classification. This problem can be tackled by reusing the feature extraction part (transfer learning) of a popular pretrained network from a very large dataset and retraining the classification part on multiple TL schemes and datasets [4]. However, transferring the pretrained weights and bias through the fixed feature extractor scheme directly to an SVM classifier also leads to the long time and memory requirements needed for training with large datasets.

Nalepa and Kawulok [47] stated that it “may be even impossible to train the classifier using a dataset encompassing a very large number of vectors”. So, the size of the training datasets may be reduced to generate a small number of support vectors, making the training phase much faster and practicable. To solve this issue, reduced augmentation data schemes were used to train the multiclass SVM on the second set of experiments. So, the data augmentation applied on the pre-processed datasets #8 to #10 was significantly reduced for (i) a one-pixel image translation with F100, (ii) F64 and a horizontal reflection image transformation, and (iii) F16, Mirror and a rotation transformation in a range of angles between −5° and +5° with a gap of 1° respectively. 

The proposed system for grape variety identification highlights some issues and constraints concerning the training phase of a deep learning network, including a very low volume of images; images captured in natural environment; significant changes on the images of grapevine leaf or bunches of grapes in different harvest seasons, mainly due to adverse climatic conditions, pests, diseases, and pesticides on the grapevines; high similarity of the images on the different grape varieties in the Douro Demarcated Region; and issues on the harvesting (both in manual or robotic) at the Douro vineyards due to the existence of more than one variety per parcel and even for row. 

Applying the TL methodology, a set of experiments comprising 10 pre-processed datasets associated with four distinct image processing techniques, three data augmentation methods, and three different AlexNet-based TL schemes allowed us to conduct an effective study of the behavior of the pretrained AlexNet model with the aim of identification of grape varieties.

According to good practice principles, our experimental results should be compared with some other authors’ results for system validation purposes. Taking into account that the natural image databases were constructed during this investigation and the deep classifier presented in this paper identified the grape varieties at the Douro Demarcated Region, it became impossible to make comparisons with other authors because no other authors the same image databases or identified any kind of grape varieties at this viticulture region.

Nevertheless, we compared our results with other results obtained by methods for identifying plant species from leaf images. The proposed system was tested on the publicly available Flavia leaf dataset, which contains 1907 images of 32 different plant species. Each species has 50 to 77 sample leaves. Each image has a resolution of 1600 × 1200 pixels on white background. These images were pre-processed with the four-corners-in-one procedure and trained using the “Learning on last 3 conv. layers” TL scheme. For each type of plant in the Flavia dataset and before the training phase, 10 species of leaves from the dataset were randomly removed, which were then used to test the performance and efficiency of the proposed system. A classification accuracy of 89.75%, trained on the pre-processed Flavia dataset with the proposed four-corners-in-one image warping method augmented with F121 (11 × 11) and mirrored images, was reached.

Comparing the classification results on the Flavia dataset with the work performed by other authors, it can be seen that Satti et al. [48] presented an accuracy of 85.9% and 93.3% for KNN and ANN classifiers, respectively, while Zhang et al. [49] presented a table comparing the accuracy values with 13 other author’s schemes (on the same Flavia dataset). The accuracy average of the works presented in that table was 87.37%. In the paper by Barrientos [50], the AlexNet type of Philip Xue obtained the best accuracy in every dataset, reaching a 91% accuracy running on 84 epochs. The reported accuracy values were in the same order of magnitude, or even lower than that obtained by the proposed system for the grape variety identification.

Regarding the high similarity of the leaf images on the different species studied, Kho et al. [15] identified only three leaf species of Ficus (among 1000 species worldwide) which hae similar leaf morphology. Their proposed system to recognize leaf images reached an accuracy of 83.3%. Compared with our approach, an accuracy score of 77.30% to identify six grape varieties was reached, i.e., twice the distinct number of varieties as reported in this paper.

## 5. Conclusions and Ongoing Work

Automatic grape variety identification is a matter of great interest in precision viticulture. Traditionally, the Douro Region vineyards produce different grape varieties in the same parcel, evidencing high visual similarity between different grape varieties, making their identification a challenging task, even for viticulture experts.

In this paper, an approach based on the AlexNet architecture with transfer learning scheme was presented to automatically identify and classify six grape varieties that predominate the Douro Demarcated Region, which can be applied, for example, in robotic/automated harvesting contexts. The computer can automatically classify six kinds of grape varieties via the segmented vine leaf images. Image pre-processing and data augmentation were adopted to reduce the overfitting degree of the model. The proposed four-corners-in-one image warping method has become the most relevant IP technique and was applied on the generation of the pre-processed datasets for the automatic identification of grape varieties in natural images using different TL schemes over a pretrained AlexNet architecture, as presented in Table 6. 

The experimental results demonstrated the reliability of the proposed classifier with a testing accuracy of 77.30%. The computation time to identify the grape variety in an image was about 6.1 ms. Applying the same classifier model, an accuracy of 89.75% on the popular Flavia leaf dataset was achieved. These results are promising and certainly encouraging, showing that the proposed approach may be an effective solution, which is believed to outperform the manual recognition of a viticulturist expert. 

For future work, our prime suggestion is related to the acquisition of a higher volume of natural images captured in different geographical locations and harvest seasons at the vineyards of the Douro Demarcated Region for training purposes. As a second suggestion, comparative evaluation of some extremely deepest networks for improvement of the classification accuracy should be done. Different TL schemes on deepest models should be tested, such as the VGG net, Inception V4, Resnet (50,101 and 152 layers), and Densenet.

For a new future implementation, we suggest definining different learning rate values for each convolutional layer, because the results obtained on dataset #8–#10 show that the TL scheme “Learning on last 3 conv. layers” performed better than changing all the weights within the whole architecture (i.e., on the “FE and classification part” TL scheme).

Given the promising results reported in this research, the proposed four-corners-in-one image warping method should be specifically used to generate pre-processed datasets to train and test the deep networks for grape variety identification in the Douro Demarcated Region. 

## Figures and Tables

**Table 1:** Distribution of the images per grape variety captured in the 2016 and 2018 harvest seasons.

	DRGV Dataset		DRGV_2018 Dataset	
Grape Variety	No. of Images	Vineyard	No. of Images	Vineyard
*Tinta Amarela*	24	*Quinta da Pacheca*	14	*Quinta do Vallado*
*Tinta Barroca*	23	*Quinta do Vale Abraão*	14	
*Touriga Franca*	23		13	
*Touriga Nacional*	22		15	
*Tinta Roriz*	23		14	
*Tinto Cão*	25	*Quinta da Pacheca*	14	*Quinta da Pacheca*

**Table 2:** Statistics of the used pre-processed datasets from the DRGV image dataset.

Input Raw Dataset 640 × 960 × 3	Pre-Processing			Data Augmentation		Output Pre-Processed Dataset 227 × 227 × 3
	Method		N. of Images	Method	N. of Images	
DRGV	Not used		140	F900	126,000	#1
	ICF		28,000	F4	112,000	#2
	LSA + 4-Corners-in-One		140	F900	126,000	#3
	LSA +	LPE	3644	F25	91,100	#4
		LPE + ICF	3644	F25	91,100	#5
		LPE + CED	3644	F25	91,100	#6
		LPE + GMP	3644	F25	91,100	#7

**Table 3:** Data splitting from DRGV + 2018 pre-processed datasets #8 to #10.

Data Splitting	Sample Size of All Grape Varieties		
	#8	#9	#10
Augmented training/validation sets	147,764	295,364	360,964
Original raw image set	164	164	164
1-pixel image translation	147,600	147,600	16,400
Image horizontal reflection	—	147,600	16,400
Image rotation	—	—	328,000
Augmented test set	54,060	108,060	132,060
Original raw image set	60	60	60
1-pixel image translation	54,000	54,000	6000
Image horizontal reflection	—	54,000	6000
Image rotation	—	—	120,000
Total	201,824	403,424	493,024

**Table 4:** AlexNet deep network in DL Matlab™ toolbox (adapted from Hertel et al., 2015).

No.	Layer (Name)	Dimensions			Kernel	Stride	Padding	Observation
		Width	Height	Depth				
1	Image input	227	227	3	−	−	−	‘zerocenter’ normalization
2	Convolution (conv1)	55	55	96	11	4	0	
3	ReLU (relu1)	55	55	96	−	−	−	
4	Cross channel (norm1)	55	55	96	−	−	−	
5	Max pooling (pool1)	27	27	96	3	2	0	
6	Convolution (conv2)	27	27	256	5	1	2	
7	ReLU (relu2)	27	27	256	−	−	−	
8	Cross channel (norm2)	27	27	256	−	−	−	
9	Max pooling (pool2)	13	13	256	3	2	0	
10	Convolution (conv3)	13	13	384	3	1	1	
11	ReLU (relu3)	13	13	384	−	−	−	
12	Convolution (conv4)	13	13	384	3	1	1	
13	ReLU (relu4)	13	13	384	−	−	−	
14	Convolution (conv5)	13	13	256	3	1	1	
15	ReLU (relu5)	13	13	256	−	−	−	
16	Max pooling (pool5)	6	6	256	3	2	0	
17	Fully-connected (fc6)	1	1	4096	−	−	−	Input size: 9216
18	ReLU (relu6)	1	1	4096	−	−	−	
19	Dropout (drop6)	1	1	4096	−	−	−	Probability: 0.5
20	Fully-connected (fc7)	1	1	4096	−	−	−	Input size: 4096
21	ReLU (relu7)	1	1	4096		−	−	
22	Dropout (drop7)	1	1	4096	−	−	−	Probability: 0.5
23	Fully-connected (fc8)	1	1	1000	−	−	−	Input size: 4096
24	Softmax (prob)	1	1	1000	−	−	−	
25	Classification (output)	1	1	1000	−	−	−	‘crossentropyex’ with 1000 classes

**Table 5:** Training, validation and test accuracy, and loss for different transfer learning schemes on the AlexNet-based network (DRGV pre-processed datasets).

TL Scheme	Metric	Pre-Processed Dataset						
		#1	#2	#3	#4	#5	#6	#7
FE and classification part	Train. Acc. [%]	100	87.50	100	65.63	56.25	31.25	37.50
	Train. Loss	0.0140	0.4511	0.0210	1.0008	1.2451	1.8715	1.3313
	Val. Acc. [%]	78.35	54.40	68.00	47.94	36.13	33.18	41.33
	Val. Loss	0.8044	1.3266	1.2182	1.3558	1.6020	1.6732	1.4717
	Test Acc. [%]	73.13	60.84	66.05	56.06	45.79	29.95	46.09
Learning on last 3 conv. layers	Train. Acc. [%]	100	87.50	100	65.63	62.50	34.38	40.63
	Train. Loss	0.0069	0.4326	0.0115	1.0936	1.1414	1.5786	1.4623
	Val. Acc. [%]	73.09	58.17	70.67	46.28	34.34	28.06	42.14
	Val. Loss	0.9551	1.3159	1.2511	1.4257	1.6374	1.7160	1.4938
	Test Acc. [%]	73.37	59.76	71.81	51.33	43.46	27.18	46.10
FE and SVM	Train. Acc. [%]	59.67	61.23	58.07	47.27	38.87	24.51	44.21
	Train. Loss	0.4033	0.3877	0.4193	0.5273	0.6113	0.7549	0.5579
	Val. Acc. [%]	68.30	43.03	47.71	39.88	33.78	25.93	36.76
	Val. Loss	0.2999	0.5799	0.5126	0.5776	0.6297	0.7366	0.6370
	Test Acc. [%]	73.43	47.98	55.74	48.14	36.62	23.94	39.25

**Table 6:** Training, validation and test accuracy, and loss for different TL schemes on the AlexNet-based network (DRGV + 2018 pre-processed datasets).

TL Scheme	Metric	Pre-Processed Dataset					
		#8		#9		#10	
		Result	Epoch	Result	Epoch	Result	Epoch
FE and classification part	Train. Acc. [%]	100	9	100	10	100	9
	Train. Loss	0.0166		0.0188		0.0171	
	Val. Acc. [%]	75.29		75.74		76.87	
	Val. Loss	0.5864		0.6248		0.7941	
	Test Acc. [%]	68.43		72.58		74.42	
Learning on last 3 conv. layers	Train. Acc. [%]	100	30	100	17	100	26
	Train. Loss	0.0110		0.0122		0.0109	
	Val. Acc. [%]	77.61		76.32		71.98	
	Val. Loss	0.6375		0.6456		0.7762	
	Test Acc. [%]	69.23		77.30		76.22	
FE and SVM	Train. Acc. [%]	83.03	n/a	83.93	n/a	76.68	n/a
	Train. Loss	0.1670		0.1577		0.2283	
	Val. Acc. [%]	47.66		46.38		47.35	
	Val. Loss	0.5237		0.5371		0.5270	
	Test Acc. [%]	52.24		58.48		53.90	

**Table 7:** Confusion matrix over the test set (in percentages).

	*Amarela*	*Barroca*	*Cão*	*Franca*	*Nacional*	*Roriz*
*Amarela*	**85.7**	0.31	0.03	0	10.06	3.9
*Barroca*	9.82	**83.3**	0	0	0.02	6.86
*Cão*	9.57	16.73	**72.95**	0.09	0	0.66
*Franca*	7.39	0.18	0	**89.1**	2.98	0.35
*Nacional*	0.43	0.79	2.73	18.86	**65.65**	11.54
*Roriz*	10.08	0.86	0	18.97	2.99	**67.1**
